# Cost-effectiveness of biennial screening for diabetes related retinopathy in people with type 1 and type 2 diabetes compared to annual screening

**DOI:** 10.1007/s10198-020-01191-y

**Published:** 2020-05-08

**Authors:** Rebecca L. Thomas, Thomas G. Winfield, Matthew Prettyjohns, Frank D. Dunstan, Wai-Yee Cheung, Philippa M. Anderson, Rajesh Peter, Stephen D. Luzio, David R. Owens

**Affiliations:** 1 Diabetes Research Unit Cymru, Swansea University Medical School, Singleton Park, Swansea, SA2 8PP UK; 2 Health Technology Wales, 3 Assembly Square, Cardiff, CF10 4PL UK; 3 Institute of Primary Care and Public Health, Cardiff University, Heath Park, Cardiff, CF14 4XN UK; 4 Swansea Centre for Health Economics, College of Human and Health Sciences, Swansea University, Singleton Park, Swansea, SA2 8PP UK; 5 Swansea Bay University Health Board, Neath Port Talbot Hospital, Baglan Way, Port Talbot, West Glamorgan SA12 7BX UK

**Keywords:** Diabetic retinopathy, Screening, Economic impact, Cost-utility analysis, I120

## Abstract

**Objective:**

Examine the health and economic impact of extending screening intervals in people with Type 2 diabetes (T2DM) and Type 1 diabetes (T1DM) without diabetes-related retinopathy (DR).

**Setting:**

Diabetic Eye Screening Wales (DESW).

**Study design:**

Retrospective observational study with cost-utility analysis (CUA) and Decremental Cost-Effectiveness Ratios (DCER) study.

**Intervention:**

Biennial screening versus usual care (annual screening).

**Inputs:**

Anonymised data from DESW were linked to primary care data for people with two prior screening events with no DR. Transition probabilities for progression to DR were estimated based on a subset of 26,812 and 1232 people with T2DM and T1DM, respectively. DCER above £20,000 per QALY was considered cost-effective.

**Results:**

The base case analysis DCER results of £71,243 and £23,446 per QALY for T2DM and T1DM respectively at a 3.5% discount rate and £56,822 and £14,221 respectively when discounted at 1.5%. Diabetes management represented by the mean HbA_1c_ was 7.5% for those with T2DM and 8.7% for T1DM.

**Sensitivity analysis:**

Extending screening to biennial based on HbA_1c_, being the strongest predictor of progression of DR, at three levels of HbA_1c_ 6.5%, 8.0% and 9.5% lost one QALY saving the NHS £106,075; £58,653 and £31,626 respectively for T2DM and £94,696, £37,646 and £11,089 respectively for T1DM. In addition, extending screening to biennial based on the duration of diabetes > 6 years for T2DM per QALY lost, saving the NHS £54,106 and for 6-12 and > 12 years for T1DM saving £83,856, £23,446 and £13,340 respectively.

**Conclusions:**

Base case and sensitivity analyses indicate biennial screening to be cost-effective for T2DM irrespective of HbA_1c_ and duration of diabetes. However, the uncertainty around the DCER indicates that annual screening should be maintained for those with T1DM especially when the HbA_1c_ exceeds 80 mmol/mol (9.5%) and duration of diabetes is greater than 12 years.

**Electronic supplementary material:**

The online version of this article (10.1007/s10198-020-01191-y) contains supplementary material, which is available to authorized users.

## Introduction

In 2019 diabetes was estimated to affect around 463 million people globally and is projected to increase to 700 million in 2045 [1]. Diabetes is a major contributor to mortality, morbidity and quality of life with an ever increasing impact on health resources [2]. In 2010/2011 the cost of diabetes to the NHS in the United Kingdom (UK) was estimated at £9.6 billion and is expected to rise to £16.9 billion by 2035/2036 [3], representing about 10% of the NHS budget with approximately 80% consumed in treating the complications of diabetes. Diabetes-related retinopathy (DR) is a feared complication of diabetes capable of causing visual impairment and severe vision loss (blindness) with devastating individual and socio-economic consequences [4–6].

DR, if undiagnosed and remains untreated in its early stages, can progress to severe visual loss. The estimated cost in the UK in 2010/2011 of treating sight-threatening DR was £57 million which is predicted to reach £97 million by 2035/2036 [3]. However, screening can detect DR well before vision is affected and when treatment is most effective to prevent progression and thereby preserve vision. To detect DR UK screening utilise digital retinal images which are then graded and according to the findings the people are reviewed annually or referred to hospital eye services for further assessment and treatment. This model in addition to possible improvements in diabetes management and newer therapies has been demonstrated to reduce new certifications for sight impairment and severe sight impairment, in Wales by 50% [7]. The proportion of sight impairment and severe sight impairment due to DR in the UK is expected to decrease from 4.7% in 2013 to 3.1% in 2050 [6]. The UK model of screening differs to that recommended in the US and elsewhere where dilated comprehensive eye examination is conducted by an ophthalmologist [8]. Although screening for DR has been shown to be cost-effective compared to no screening and offsets the costs associated with blindness [9–12], the ever increasing number of people with diabetes given annual screening may soon become financially unsustainable.

In 2016 the UK National Screening Committee also recommended that screening for DR could be increased from annual to biennial in those considered to be at low risk of progression to sight-threatening DR—following two consecutive negative annual screening events [13–18]. This recommendation is based on studies involving people only with Type 2 diabetes (T2DM) or mixed populations of Type 1 and Type 2 diabetes [13–15, 19]. However, the evidence is limited as to whether extending the screening in people with Type 1 diabetes (T1DM) is safe or cost-effective [20–26]. In 2017, the American Diabetes Association (ADA) also recommended screening be conducted every 2 years conditional on there being no evidence of DR on one or more prior annual screening events, with glycaemia well managed and with robust information technology systems and support to ensure future re-call for screening [8].

The aim of the analysis reported here is to estimate the likely economic consequences of extending screening intervals from annual to biennial in people considered to be at low risk for developing sight-threatening DR with either T1DM or T2DM. The analyses used routine clinical data taking into account type of diabetes, blood pressure (BP) and total cholesterol, and the difference in the rate of progression to DR. This will provide evidence on which regulatory authorities such as the National Institute of Clinical Excellence (NICE) and the National Screening Committee (NSC) in the UK, to base their future DR screening guidelines.

## Populations and methods

### Population and setting

People with either T1DM or T2DM (total 91,393; T1DM 5003; T2DM 86,390) undergoing screening for DR in a systematic community-based programme in Wales, UK between 2005 and 2009. Those included in the study had no evidence of DR at the first screening and had at least one further screening event [27].

Data from the Diabetic Eye Screening Wales (DESW) for all persons with diabetes ≥ 12 years registered with a GP in Wales, which included age, gender, date of diagnosis, type and treatment of diabetes, date of screening, DR grade and visual acuity [12] were transferred to the National Health Service (NHS) Wales Informatics Service for anonymisation and given a unique linking field number [28]. The data were then transferred to the Secure Anonymised Information Linkage (SAIL) databank [29] where it was linked with the Welsh Longitudinal General Practice (WLGP) dataset [30] which contained information on HbA_1c_, BP, cholesterol level, treatment for hypertension and dyslipidaemia, diabetes treatment, and smoking status. The WLGP dataset contains data from approximately 80% of primary care practices in Wales. The combination of the DESW and WLGP datasets, along with the requirement of at least 2 screening events (the first of which had no evidence of DR) with clinical information available within the WLGP database and with a confirmed specific diagnosis of T1DM or T2DM (and not ‘diabetes’), limited the population size available for the analysis. Therefore, the number of people included in the combined, final datasets available for analysis to 1232 people with T1DM and 26,812 people with T2DM.

### Transition probabilities

Information was sourced from the literature on transition probabilities for the different levels of DR whilst under the care of hospital eye services (Supplementary Table 1), treatment and service costs within hospital eye services (Table 1), cost of blindness (Table 1) and quality of life (Table 2). Transition probabilities for sight-threatening DR could not be calculated from DESW data as people are referred to hospital eye services (HES) once a sight-threatening level of DR is reached and they do not re-enter screening until treatment has stabilised, therefore, the progression of DR during monitoring and treatment within HES was unavailable for this population. To derive the transition probabilities for the CUA the time to an event of sight-threatening DR was modelled although the exact time the sight-threatening DR developed was unknown because it will have occurred between 2 screening events. Therefore, the data was interval censored, as well as right censored for those where sight-threatening DR did not occur by the last time they were screened but may occur in the future. Due to the interval censoring a Cox proportional hazards model could not be used, therefore parametric models were considered. Weibull, Gompertz and lognormal models were fitted to the data and the Akaike information criterion (AIC) method used to determine the best fit. Weibull modelling was used to estimate the probability of an event of sight-threatening DR occurring for each person, based on their clinical history in the form of a survival function S(t), that is the probability that the retinopathy event has not occurred by time (*t*). This has survival and hazard functions of the form

$$S(t) = \exp ( - \lambda t^{p} ),\;h(t) = p\lambda t^{p - 1}$$

**Table 1:** Resources used in screening and treatment of DR

Procedure/condition	Cost
Screening visit in DR screening service in Wales	£33 [25]
Hospital-based DR screening visit	£106 [33]
Optical Coherence Tomography (per scan)	£117 [33]
Focal Laser/panretinal photocoagulation laser	£131 [33]
Anti-VEGF treatment for maculopathy (drug + administration costs)	£822 (£742 + £80) [33]
Vitrectomy	£989 [33]
Sight loss (person remains living at home) per annum	£1483 [33]
Sight loss (person resident in care home) per annum	£6972 [33]

**Table 2:** RDR utility values taken from Lund et al. [30]

RDR state	EQ-5D values [30]
Pre proliferative	0.7915
Maculopathy	0.7365
Pre proliferative and maculopathy	0.7365
Easy to treat proliferative	0.7047
Easy to treat proliferative and maculopathy	0.693
High risk proliferative	0.7047
High risk proliferative and maculopathy with visual impairment	0.693
Severe proliferative	0.7047
Severe proliferative with maculopathy and vision loss	0.693
Severe loss of vision	0.6218

Weibull regression analysis was implemented by the routine INTCENS programme in STATA. Risk factors for the progression of DR were added to the Weibull model in a stepwise manor with those not statistically significant removed. The final Weibull model used to generate transition probabilities for moving from one severity level of DR to another including the risk factors HbA_1c_, BP, cholesterol level, treatment of hypertension and dyslipidaemia, diabetes treatment and smoking status (Supplementary Table 2).

**Fig. 1:**
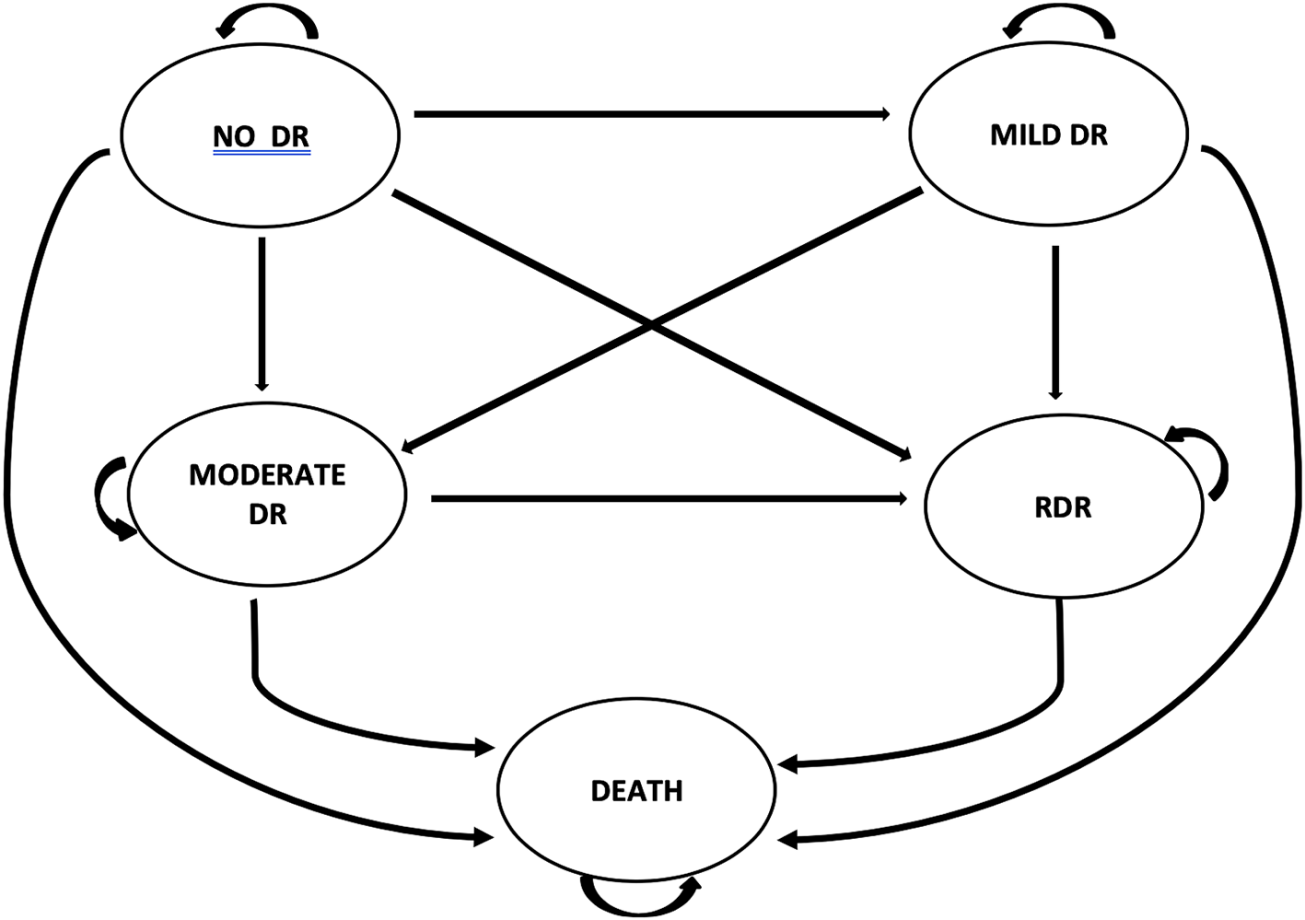
The Markov model structure

The transition probabilities derived from the survival analysis were then used to populate a de-novo multi-level Markov model to estimate the impact of extending screening beyond annual and the consequences at different HbA_1c_ levels were estimated. The model structure and flow of people are represented in Fig. 1. Life tables were used in the analysis. Life table data for the general population obtained from the Office of National Statistics (ONS) was used with adjustments made to reflect the increased mortality risk associated with T1DM or T2DM.

### Model inputs

DR in Wales is graded according to the enriched version of the English DR grading protocol [27, 31] where No DR means no lesions of DR are visible on the retina; minimum Background DR (BDR) where microaneurysms, haemorrhages and exudates (not within 1 disc diameter of the fovea) can all be present but do not meet the criteria for Moderate BDR; Moderate BDR where microaneurysms, < 8 blot haemorrhages and exudates (not within 1 disc diameter of the fovea) are present and Referable DR (RDR) consisting of Pre-proliferative DR (PPDR) where > 8 blot haemorrhages, intra-retinal microvascular anomalies (IRMA) and venous beading are present, Proliferative DR (PDR) where new vessels on the optic disc and/or elsewhere, pre-retinal haemorrhages, vitreous haemorrhages, retinal detachment and fibrosis are present and exudative maculopathy.

As the primary care dataset obtained from SAIL was related to GP appointments and not screening events, modelling methods were employed to enable estimation of the clinical values (HbA_1C_, systolic and diastolic BP, serum cholesterol, high and low-density lipids, triglycerides, GFR, body mass index (BMI)] which were combined into body mass index [BMI] at the time of DR screening. Multilevel modelling was used for the longitudinal data using a quadratic function of time for the correlation between different measurements from the same subject but constrained to follow a normal distribution across subjects. These coefficients were combined with the times of screening allowing prediction of the values at each screening event.

For the economic analysis the population and distribution of people with diabetes undergoing screening in Wales in 2005–2009, i.e. 5003 people with T1DM and 86,390 people with T2DM—were used to populate the model [27]. The economic analyses estimated the rate of progression from no disease to different stages of DR and the dynamic transition probabilities from the estimates of progression were incorporated into the model (supplementary Tables 3a and 3b).

### Comparators and time horizon

The impact of extending the screening interval to biennial was compared against usual care which is annual screening for both people with T1DM and T2DM. The analysis was conducted with a lifetime time horizon enabling estimation of the long-term impact on DR and its management resulting from changes to the screening schedule.

### Discount rate

As some of the observed DESW screening intervals exceed the target of an annual review at exactly 12 months, the future costs and benefits beyond 12 months were discounted back to present day values in line with the NICE reference case at a standard rate (currently 3.5% with sensitivity analysis at 1.5%) [32].

### Economic evaluation methods

To assess the relative cost effectiveness of extending screening intervals from annual to biennial a cost-utility analysis (CUA) was used. A Markov model was utilised to facilitate the CUA with a cycle length of 6 months. The impact on health-related quality of life (HRQoL) quantified as health utilities according to the health status characterising DR in its later stages as described by Royle and colleagues in their study were used [33].

### Outcomes

Quality-adjusted life years (QALYs) were the outcome measure used to calculate the difference in cost between screening interval options, divided by the difference in their effect. The decremental cost-effectiveness ratio (DCER), an approach used in a situation where the analysis of a health strategy appears to reduce healthcare expenditure by removing or substituting an intervention, was also calculated (supplementary Fig. 1). The decision rule is that a strategy is considered cost-effective if the incremental cost-effectiveness ratio (ICER) is below a threshold of £20,000 per QALY, as used by the National Institute of Health and Care Excellence (NICE) in England and Wales. The reverse is true for a DCER, with values above the threshold of £20,000 per QALY considered cost-effective.

### Resources and costs

An NHS and personal social services (PSS) perspective was adopted and the model was designed to estimate healthcare resource use and cost of sight-threatening DR treatment and management.

### Currency, price, date and conversion

A price year of 2018 was used in the analysis and costs were reported in pound sterling (£). A number of sources were used to identify the relevant UK NHS resources and related costs associated with DR screening, treatment and management of different stages of DR (Table 1).

### Assumptions

Analysis of our data revealed the progression of DR to be a dynamic process, i.e. the development of RDR accelerates with increasing severity of DR. To ensure the model also reflected usual practice within DESW, the model pathway was divided into four DR states i.e. No DR, Minimum BDR, Moderate BDR, and RDR. People entered the model at the time of the first screening event. Those who entered the RDR state within the first year of the 2-yearly screening interval were assumed (based on clinical advice) to progress without treatment during the following year. People who stayed within the RDR state were characterised by PDR and maculopathy.

The impact of a delay in the identification of people with RDR, because of longer screening intervals, is driven primarily by the severity of DR diagnosed at the first visit to the hospital eye service (HES) following referral from the screening service. The prevalence for people attending HES was calculated by progressing the current RDR distribution [27], in combination with the transition probabilities reported by Royle et al. [25].

On referral to HES, the RDR pathway followed in Wales incorporated ten clinically relevant health states with differing severity levels of DR and maculopathy. The assumptions made about treatment for those within the RDR category in HES can be seen in Supplementary Tables 4a and 4b [30]. The use of surrogate markers for maculopathy based on non-stereoscopic digital photography may result in over referral to HES for further assessment with a proportion discharged after the first HES appointment back to DESW. To account for this and simulate the known occurrence of false positives from the DESW screening reports and subsequent referrals to HES which prove negative, a linear reduction of one severity step within the RDR states was implemented at a rate of 50%, this adjustment was made to reflect an appropriate ‘knockback’ effect [27].

The RDR distribution was progressed through the model for one year to simulate the unidentified progression of DR (Supplementary Table 5) and related health service treatment costs and the decrement in health utility. The resulting value was adjusted to represent the average difference in cost and health utilities for a single patient within a six-month period. The combination of STDR and maculopathy and the occurrence of screening within the RDR pathway meant that patients often remained untreated for one or both conditions and were assumed to follow the same transition progression as before. People, who received treatment for STDR or maculopathy, were assumed to be ‘stationary’ within the model, thereafter.

### Sensitivity analysis

To explore the effect of uncertainty on the outcomes of our analyses we conducted both deterministic and probabilistic sensitivity analysis.

## Results

### Study parameters

People with T1DM without DR at first screening event were younger (22.7 years) with a longer duration of diabetes (7.8 years) and a higher HbA_1c_ [71.6 mmol/mol (8.7%)] compared to those with T2DM without DR [age 62.3 years, known duration of diabetes 4.2 years, HbA_1c_ 58.5 mmol/mol (7.5%)] (Table 3). All people with T1DM were treated with insulin, with 1.6% receiving metformin and surprisingly 9.8% were recorded as being prescribed sulphonylureas in addition to their insulin which casts doubt on the accuracy of the designated type of diabetes recorded. However, in the absence of being able to review the clinical data due to the anonymisation procedure no change was made to the database, but this limitation was noted.

**Table 3:** Demographics of the DESW population 2003–2013 included in the statistical analysis to estimate the transition probabilities

	T1DM (1232)	T2DM (26,812)
Age mean (SD)	22.7 (11.6)	62.3 (12.1)
Gender: male %	53.8	56.1
Known duration of diabetes mean (SD)	7.8 (9.4)	4.2 (4.1)
HbA_1c_ (mmol/mol) mean	71.6	58.5
HbA_1c_ (%) mean (SD)	8.7 (1.6)	7.5 (1.2)
*Treatment of diabetes %*		
Metformin (as one treatment)	1.6	60.5
Sulphonylurea (as one treatment)	9.8	22.0
Insulin alone	88.5	4.1
Systolic BP mean (SD)	121.7 (9.5)	138.2 (10.6)
Diastolic BP mean (SD)	71.8 (5.6)	79.2 (6.3)
Total cholesterol	4.5 (0.7)	4.6 (0.8)
HDL-cholesterol	1.5 (0.4)	1.2 (0.3)
LDL-cholesterol	2.5 (0.6)	2.4 (0.7)
Triglycerides	1.3 (0.6)	2.1 (1.0)
eGFR	107.9 (22.8)	79.5 (18.7)
BMI mean (SD)	24.1 (4.6)	32.3 (6.3)
Smokers: yes %	3.8	38.7

Transition through the stages of DR to RDR occurred faster for people with T1DM compared to those with T2DM. The risk of progression analysis indicates that HbA_1c_ and duration of diabetes were the strongest predictors of progression of DR. The transition probabilities were highly sensitive to the threshold level of HbA_1c_ for both T1DM and T2DM, with higher levels resulting in an increased rate of DR progression. Supplementary Figs. 2a, 2b, 3a and 3b illustrate the time to the development of RDR for T1DM and T2DM at different levels of HbA_1c_ and known duration of diabetes. The median time to the development of RDR occurred at 20 years for HbA_1c_ of 47.5 mmol/mol (6.5%), 13 years at 63.9 mmol/mol (8.0%) and 8 years 74.9 mmol/mol (9.0%) respectively for T1DM and > 20 years, 17 years and 12 years respectively for T2DM. For people with T1DM there was a distinctly slower rate of progression to RDR for those with a shorter duration of diabetes (< 6 years) versus those with a longer duration (≥ 6 years) with little difference in the speed of progression between 6–12 and > 12 years duration. For people with T2DM the speed of progression to RDR was quickest in people with diabetes for > 6 years, intermediate for 3-6 years and slowest for < 3 years duration. The median time to development of RDR for T1DM was < 20 years after < 6 years of diabetes, and 10 years after ≥ 6 years of diabetes. In comparison, the median time to development of RDR in T2DM was > 20 years with a known duration of diabetes of < 3 years, 19 years after 3–6 years and > 15 years after a known duration of diabetes of > 6 years.

### Incremental costs and outcomes

The base case results of the analysis showed that extending the screening interval in people with T1DM and T2DM to biennial reduced costs by £37 and £79 per person, respectively and effectiveness by 0.0016 and 0.0011 QALYs respectively. The resulting DCERs of £23,446 and £71,243 per QALY for T1DM and T2DM, respectively are above the threshold of £20,000 per QALY indicating that the biennial screening strategy is cost-effective in both populations.

### Sensitivity analysis

Deterministic sensitivity analysis was conducted for a number of scenarios using the strongest predictors of progression to RDR i.e. HbA_1c_ and known duration of diabetes (Table 4). For people with T1DM at Analysis 2: HbA_1c_ levels of 48 mmol/mol (6.5%) the DCER was £94,696 (analysis 1) falling to £37,646 at 64 mmol/mol [8.0% (analysis 2)], to £23,446 at 72 mmol/mol [8.7% (analysis 3)] and finally to £11,089 at 80 mmol/mol [9.5% (analysis 4)]. When the duration of diabetes was varied those with T1DM for < 6 years had a DCER of £83,856 (analysis 5) which reduced to £23,446 after 6-12 years (analysis 6) and £13,340 after 12 years (analysis 7). These analyses, at a threshold of £20,000, suggest that for those people with T1DM for less than 6 years it would be considered cost-effective to extend the screening interval to biennial (analysis 5), becoming borderline cost-effective between 6 and 12 years (analysis 6) and not cost-effective after 12 years (analysis 7) nor would it be cost-effective at a HbA_1c_ level between of 80 mmol/mol (9.5% [analysis 4]) or above. In contrast for those people with T2DM extending the screening interval to biennial for all analysis performed the DCER remained cost effective at a threshold of > £20,000 for all modelled HbA_1c_ levels and diabetes durations.

**Table 4:** Estimated DCERs for increasing annual screening to biennial screening by HbA_1c_ level and duration of diabetes

	HbA_1c_ level mmol/mol (%)				Duration of diabetes (years)		
	48 (6.5)	64 (8.0)	72 (8.7)^a^	80 (9.5)	< 6	6–12	> 12
T1DM (DCER QALY £)	94,696^c^	37,646^c^	23,446^c^	11,089	83,856^c^	23,446	13,340

	HbA_1c_ level mmol/mol (%)				Duration of diabetes (years)		
	48 (6.5)	58.8 (7.5)^b^	64 (8.0)	80 (9.5)	< 3	3–6	> 6
T2DM (DCER QALY £)	106,075^c^	71,243^c^	58,653^c^	31,626^c^	87,405^c^	71,243^c^	54,106^c^

^a^Mean HbA_1c_ value for the population with T1DM

^b^Mean HbA_1c_ value for the population with T2DM

^c^Changing to biennial screening ICER considered cost effective at above £20,000 per QALY lost threshold

Additional sensitivity analysis was performed where the discount rate for costs and outcomes was reduced to 1.5%. This reduction reduced the DCER for biennial screening intervals to £14,221 per QALY lost for T1DM and £56,822 per QALY lost for T2DM.

Probabilistic sensitivity analysis was conducted to assess the combined parameter uncertainty in the model. In this analysis, the mean values that were utilised in the base-case were replaced with values drawn from distributions around the mean values. At a threshold of > £20,000 per QALY gained, extending the screening interval to biennial was found to have a 57% and 97% probability of being cost-effective in people with T1DM and T2DM, respectively.

## Discussion

Our analysis suggests that for the base case at the discount rate of 3.5% extending the screening interval from annual to biennial for people with T2DM and T1DM without evidence of DR at first screening exceeded the threshold for cost-effectiveness at £71,243 and £23,446 respectively. Therefore, increasing the screening interval achieves a substantial resource saving with only a very small impact on the outcomes. However, if the discounted rate was reduced to 1.5% increasing the screening interval remained cost-effective for those with T2DM at £56,822, but not for those with T1DM at £14,221 for whom screening should continue annually.

As glycaemic control (HbA_1c_) and duration of diabetes are known key risk factors for the onset and progression of diabetic retinopathy they were therefore included in a sensitivity analysis at the 3.5% discount rate. In those with T2DM the HbA_1c_ levels and duration of diabetes did not reduce the cost-effectiveness when extending the screening interval from annual to biennial. However, for those with T1DM extending the screening interval beyond annual was only cost-effective when the HbA_1c_ was below 64 mmol/mol (8.0%) and was only borderline cost effective at 72 mmol/mol (8.7%) and clearly not cost effective at 80 mmol/mol (9.5%) or higher. Also extending the screening intervals was only cost-effective when the duration of T1DM was ≤ 12 years.

Probabilistic sensitivity analysis demonstrated that the degree of uncertainty around the result was very different in people with T2DM and T1DM. There was relatively little uncertainty around the result in T2DM with a 97% probability, suggesting that the recommendation of extending the screening interval to biennial would very likely be cost-effective. Conversely, there was a high degree of uncertainty around the result in T1DM with a 57% probability that extending the screening interval to biennial would be cost-effective and, therefore, not a strategy to be recommended for any T1DM population based on our findings.

It is important to note that the outcomes of our analyses are based on a ‘perfect health system’ where all screening resources and costs that are recoverable can be deployed elsewhere and screening uptake is assumed to be 100%. Important potentially quantifiable factors not available to include in this analysis, since they are setting specific, are the extent of the estimated resource and cost savings recoverable from increasing screening intervals if the increase in screening intervals were implemented for the groups of people identified in this analysis. Also influential are the ascertainment rates of the population to be included in the screening programme [34], as well as uptake rates for the screening programme which in Wales is currently 80% [35]. These factors are likely to reduce the borderline cost-effectiveness, based at the 3.5% discount rate, for T1DM as well as those in the sensitivity analysis at HbA_1c_ level of 72 mmol/mol (8.7%) and 6–12 years duration of diabetes to below the threshold of greater than £20,000 considered to be cost-effective by NICE.

An important driver of outcomes in the model is the increased risk of progression to RDR, which influences the outcome for the person with DR if there is a delay in treatment due to screening interval increases. The speed of transition and missed treatment opportunities for people with RDR consequently increases the number of people requiring treatment, thus increasing health care resource use and costs. Another issue for outcomes is that over the time horizon of the model people with T1DM experience a higher mortality rate than those with T2DM [24]. On the other hand, people with T2DM are older at the onset of their diabetes, have a shorter exposure to diabetes but are more likely to die within the time horizon of our model as a result of a shorter remaining lifespan.

Previous studies that have assessed the cost effectiveness of extending screening intervals have either focussed solely on people with T2DM [22, 23] or have combined people with T1DM and T2DM into one group [10, 25] and found that increasing screening intervals to 2-5 years to be safe and cost effective in those at low risk of progression to sight-threatening DR. The DCCT/EDIC study group did look exclusively at people with T1DM [33] however this population was from a Randomised Control Trial and so generalisability to real-world populations is limited. This was based on a 4 year, 3 year, 6 month and 3 month screening schedule for those participants of the DCCT/EDIC trial categorised into low to high-risk groups resulting in a 58% reduction in screening appointments per patient versus annual screening and projected to save approximately $1 billion over a 20 year period. Eleuteri et al. developed a risk calculation engine (RCE) to assign personalised screening intervals and found that individualised intervals of 6, 12 and 24 months with a 2.5% risk threshold using the RCE method was feasible, reliable, safe and acceptable to people with diabetes whilst reducing the number of screening episodes by 30% [36]. Although the RCE used covariates such as duration of diabetes, HbA_1c_, BP and total cholesterol but unfortunately the type of diabetes was not included and no adjustment was made for the difference in the rate of progression of DR between T1DM and T2DM.

Wales has a higher prevalence of diabetes than the remainder of the UK [37]. Also screening models differ slightly in that England employs a mixture of programmes using optometrists or screener graders and Scotland predominantly rely on only one retinal image per eye. However, these findings should still be applicable where systematic screening programme exist with central call/recall facilities with the possible exception of those regions or countries with higher proportions of ethnic minorities which have a higher risk of diabetes and progression of complications [38].

We acknowledge that there are limitations to this study related to the data recorded by DESW when people with diabetes first appear for their first screening event following a referral from general practice. Data cleaning procedures were employed to ensure the accuracy of the diagnosis of diabetes, despite which a proportion of people classified as T1DM were reported to be treated both with insulin and with metformin (1.6%) and/or sulphonylureas (9.8%) which indicate that this small cohort of participants could well be miss-classified, but this could not be confirmed due to the anonymisation procedure. This potential ‘contamination’ is unlikely to have affected the results for people with T1DM such that our recommendations would change. In addition, the exclusion of comorbidities could also be a limitation.

The modelling methods used to estimate the impact of changing the screening interval include uncertainty inherent in the evidence informing the model, such as the natural history of DR progression and the association between health utility scores and visual acuity and the impact on costs and outcomes of the various treatments. We were unable to source from the local service data some transition probabilities for variables e.g. those related to HES, treatment effectiveness, health utility scores and the economic consequences of visual loss which were therefore obtained from the literature. As a result, albeit that these transition probabilities were derived from UK data, the populations on which these studies were based mean that their results might be different from that which the majority of our data were obtained.

The strength of this study is that it is the first large community based nationally representative dataset that links person-level data on DR screening and risk factors for the progression of DR. Importantly the addition of clinical diagnoses and risk factors to the dataset allowed analysis to be conducted separately for T1DM and T2DM. There have been very few studies to date of the cost-effectiveness of extending the screening interval particularly in people with T1DM alone.

Our analyses, reported here, identified a distinct difference in relative cost effectiveness at different thresholds of HbA_1c_ and duration of diabetes, driven by the underlying risk of progression to RDR between people with T2DM and T1DM. This means that extending screening intervals to biennial in people with T2DM is most likely to be cost effective according to current NICE guidelines which would allow policy makers to re-deploy the gains elsewhere. However, extending screening intervals beyond annual for people with T1DM appears only to be clearly cost effective when HbA_1c_ level is < 64 mmol/mol (< 8.0%), and duration of diabetes < 6 years. Extending the screening interval in persons with T1DM beyond annual should not be introduced where HbA_1c_ is above 80 mmol/mol (9.5%) and the duration of diabetes is greater than 12 years.

Ultimately screening programmes should adopt the principles of precision medicine requiring further research to arrive at an algorithm that can individualise screening intervals of based on the current status of the retinal vasculature and the presence of putative risk factors thereby optimising the use of resources without increasing the risk of vision loss and blindness.

## Electronic supplementary material

Below is the link to the electronic supplementary material.

Supplementary material 1 (DOCX 829 kb)
