# Supplementary material for: Cost-effectiveness of biennial screening for diabetes related retinopathy in people with type 1 and type 2 diabetes compared to annual screening
Source: Eur J Health Econ. 2020 May 8;21(7):993–1002. doi: 10.1007/s10198-020-01191-y (PMC7423794; doi:10.1007/s10198-020-01191-y)
Supplement: Supplementary file 1 — Supplementary material 1 (DOCX 829 kb) [file 10198_2020_1191_MOESM1_ESM.docx]

Title: Cost-effectiveness of biennial screening for diabetes related retinopathy in people with type 1 and type 2 diabetes compared to annual screening

European Journal of Health Economics

Appendix components

Interpreting an incremental cost effectiveness ratio (ICER) can be facilitated using a*cost-effectiveness* plane (Figure 1) where ICERs are presented graphically as a combination of the costs and the effects of a health intervention, compared to the alternative (in this case the comparison is being made between differing screening intervals). Costs are conventionally placed on the north-south axis and effects on the east-west axis. The dotted diagonal line marks a threshold above which the ICER no longer represents perceived value for money. In the south-east quadrant of the cost effectiveness plane the intervention, compared with the alternative is cost saving (lower costs and more effective than the alternative) while in the north-east quadrant an intervention will not be acceptable as it costs more and is less effective than the alternative. In the south west quadrant if the ICER falls below the threshold, the intervention has lower costs and less effective than the alternatives. The ICER in this case refers to a cost saving per unit of effect lost. The commonly assumed decision rule of accepting ICERs below a given threshold in the south west quadrant is reversed and the higher the ICER the more cost effective the treatment becomes.

**Figure 1: The cost effectiveness plane**


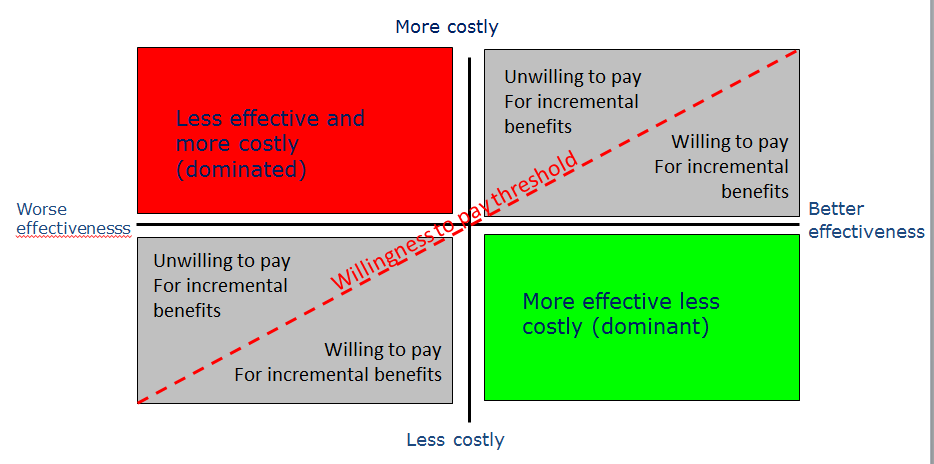


Figure 2: Incidence of RDR by different HbA_1c_ levels in people with a) type 1 diabetes and b) T2DM

a) type 1 diabetes b) T2DM

HbA_1c_ level

Supplementary Figure 3: Incidence of RDR by duration of diabetes in people a) type 1 diabetes and b) type 2 diabetes

a) type 1 diabetes b) type 2 diabetes

Duration of diabetes (years)

Supplementary Figure 4: Incremental cost-effectiveness ratio (ICER) scatterplot for probabilistic sensitivity analyses in population with type 1 diabetes

The results of the probabilistic sensitivity analysis (PSA) for the population with type 1 diabetes are shown using an ICER scatterplot, which shows the incremental costs and QALYs associated with each of the 10,000 runs of the PSA along with the mean result. It can be seen that the majority of results reside in the south west quadrant of the diagram indicating that extending the screening interval to two years is less effective and less costly in most modelled scenarios.


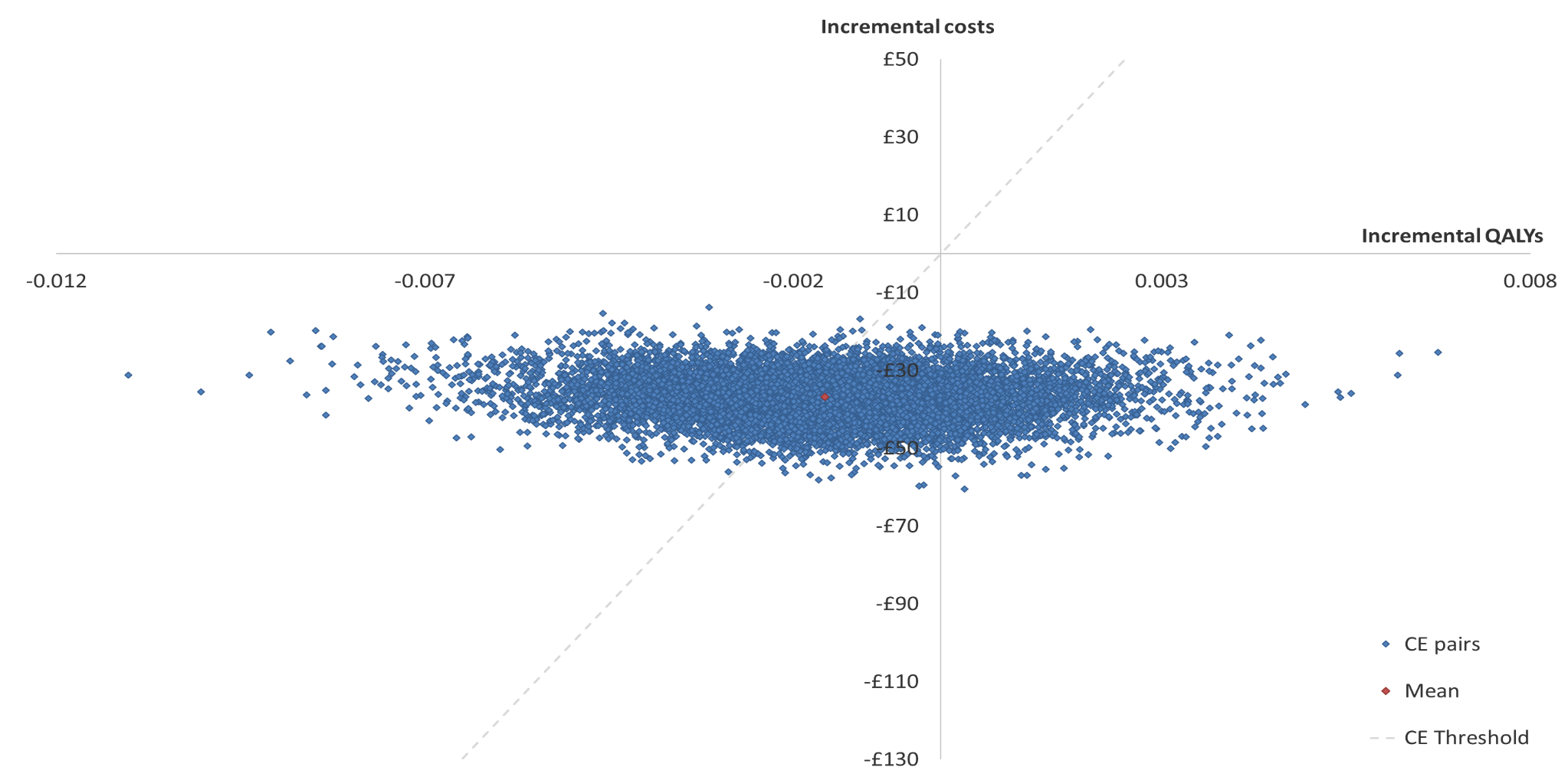


Supplementary Figure 5: Incremental cost-effectiveness ratio (ICER) scatterplot for probabilistic sensitivity analyses in population with type 2 diabetes

The results of the probabilistic sensitivity analysis (PSA) for the population with type 2 diabetes are shown using an ICER scatterplot, which shows the incremental costs and QALYs associated with each of the 10,000 runs of the PSA along with the mean result. It can be seen that the majority of results reside in the south west quadrant of the diagram indicating that extending the screening interval to two years is less effective and less costly in most modelled scenarios.


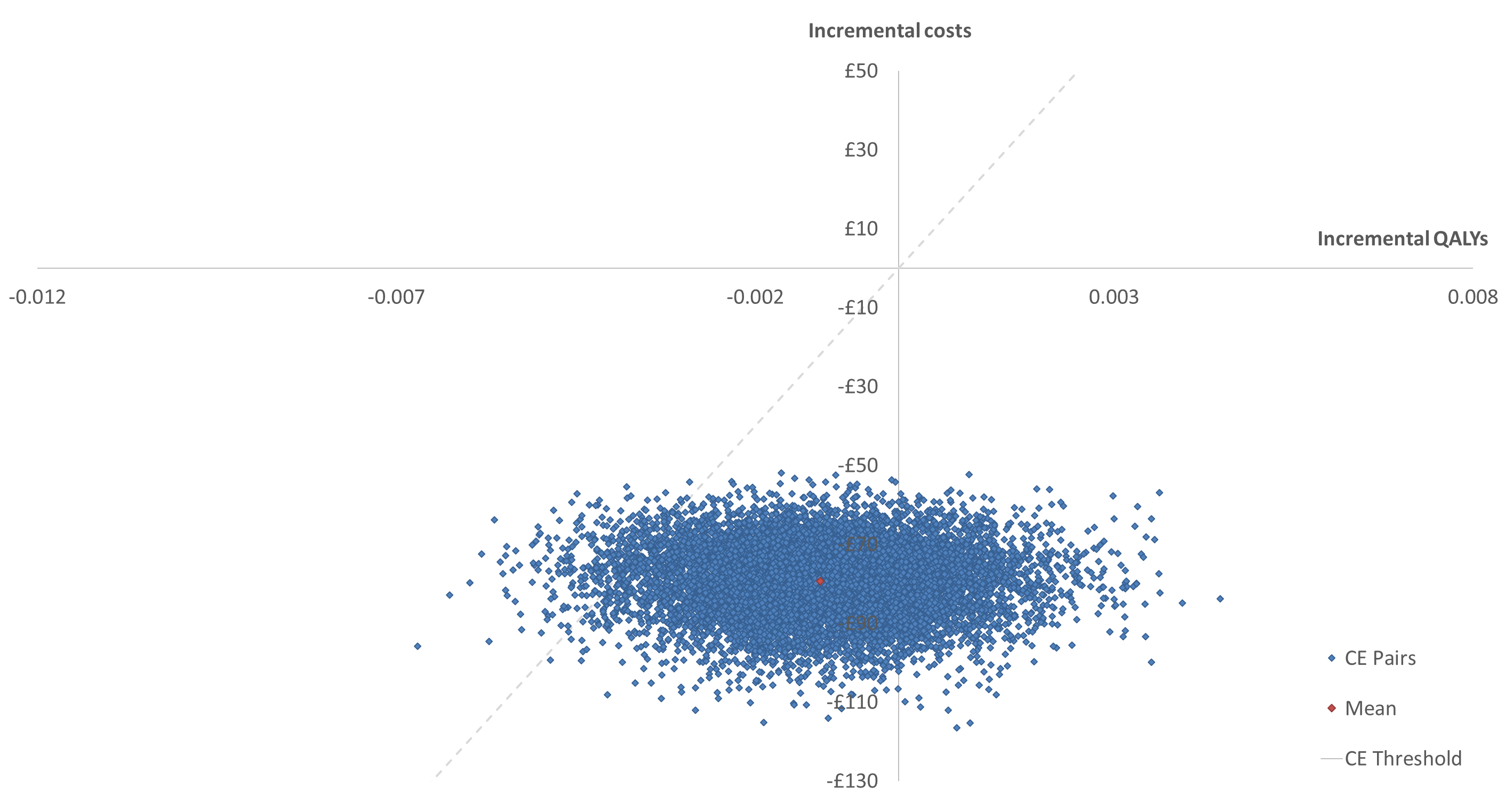


Supplementary Figure 6: Cost-effectiveness acceptability curves (CEACs) for probabilistic sensitivity analyses in population with type 1 and type 2 diabetes

The results of 10,000 runs of the probabilistic sensitivity analysis (PSA) for the population with type 1 and type 2 diabetes are shown using cost-effectiveness acceptability curves (CEACs). The curves show the probability of extending the screening interval to two years being considered cost-effective at various cost-effectiveness thresholds on the x axis. At a threshold of £20,000 per QALY, extending the screening interval to biennial was found to have a 57% and 97% probability of being cost-effective in people with T1DM and T2DM, respectively.


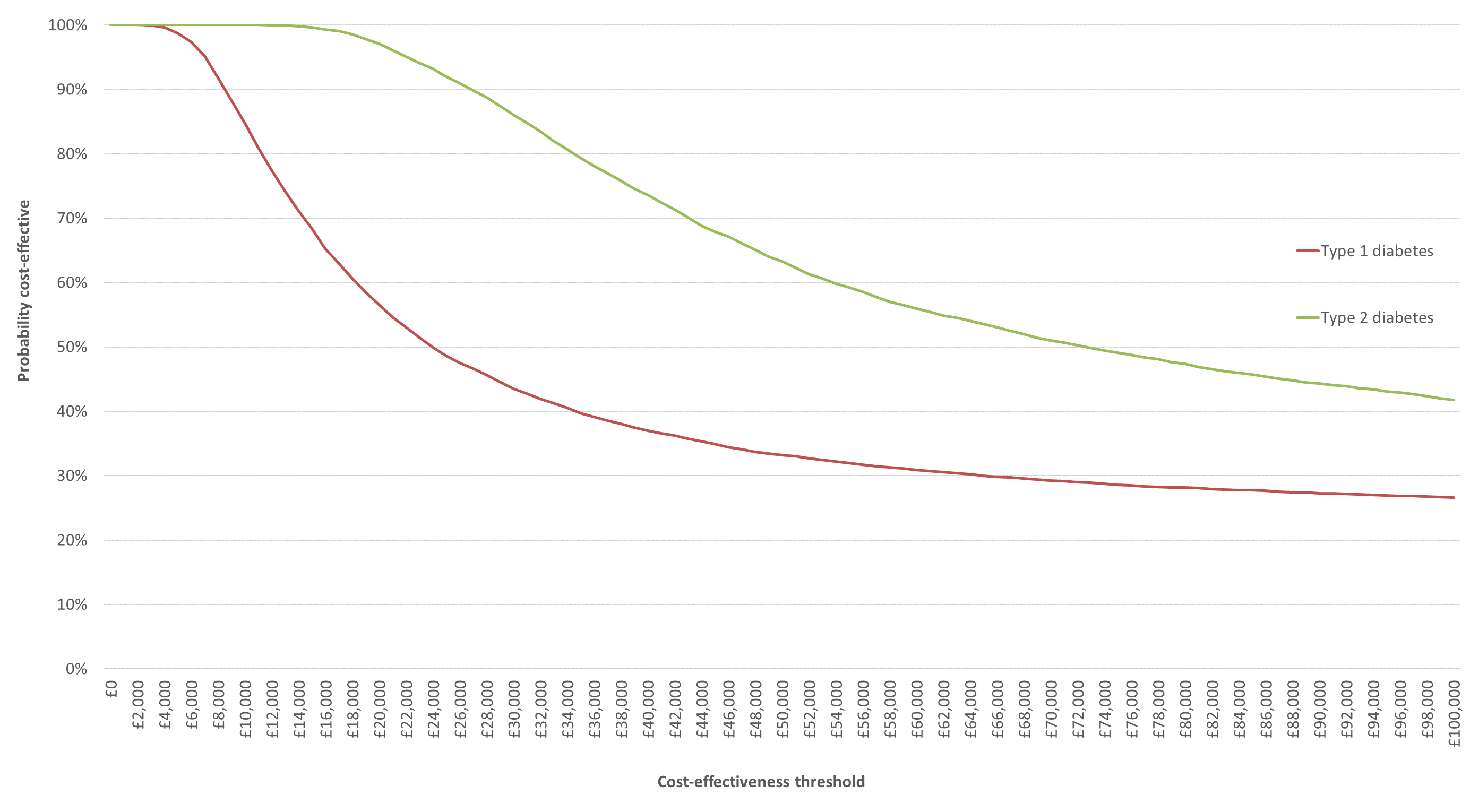


Supplementary Table 1: Transition probabilities within HES^30^

| Pre proliferative | 0.9725 | 0 | 0.0061 | 0.0214 | 0 | 0 | 0 | 0 | 0 | 0 |
| --- | --- | --- | --- | --- | --- | --- | --- | --- | --- | --- |
| Maculopathy | 0 | 0.9786 | 0.0214 | 0 | 0 | 0 | 0 | 0 | 0 | 0 |
| pre proliferative and Maculopathy | 0 | 0 | 0.9452 | 0 | 0.0548 | 0 | 0 | 0 | 0 | 0 |
| Easy to treat proliferative | 0 | 0 | 0 | 0.8444 | 0.0061 | 0.0717 | 0.0778 | 0 | 0 | 0 |
| easy to treat proliferative and maculopathy | 0 | 0 | 0 | 0 | 0.6753 | 0.1434 | 0.1555 | 0 | 0 | 0.0258 |
| High risk Proliferative | 0 | 0 | 0 | 0 | 0 | 0.896 | 0.0061 | 0.0459 | 0.052 | 0 |
| High risk Proliferative and maculopathy with Visual impairment | 0 | 0 | 0 | 0 | 0 | 0 | 0.7508 | 0.0918 | 0.1039 | 0.0535 |
| Severe Proliferative (vitrectomy) | 0 | 0 | 0 | 0 | 0 | 0 | 0 | 0.9404 | 0.0061 | 0.0535 |
| Severe proliferative with maculopathy and vision loss | 0 | 0 | 0 | 0 | 0 | 0 | 0 | 0 | 0.9465 | 0.0535 |
| Blindness | 0 | 0 | 0 | 0 | 0 | 0 | 0 | 0 | 0 | 1 |

Supplementary Table 2: Transition probabilities for progression of DR within DESW for the risk factors included for those with a) type 1 diabetes

|  |  | No DR to Mild DR | No DR to Mod DR | No DR to RDR | Mild DR to Mod DR | Mild DR to RDR | Mod DR to RDR |
| --- | --- | --- | --- | --- | --- | --- | --- |
| Constant |  | -8.788 | -13.086 | -19.354 | -15.2175 | -16.682 | -13.058 |
| Power |  | 1.086 | 1.406 | 1.588 | 2.403 | 1.606 | 0.903 |
| HbA1C |  | 0.2226 | 0.3682 | 0.5796 | 0.1437 | 0.5793 | 0.3796 |
| SBP |  |  |  |  |  |  |  |
| DBP |  |  |  |  |  |  | 0.0397 |
| BMI |  | 0.0200 | 0.0409 | 0.0598 |  |  |  |
| Time since diagnosis | <6 | 0 | 0 | 0 | 0 | 0 | 0 |
|  | 6 – 12 | 0.7092 | 0.8237 | 1.072 | 0.7597 | 0.6972 | 1.6270 |
|  | >12 | 0.8375 | 0.9718 | 1.599 | 0.0462 | 1.2231 | 1.5439 |
| Smoking status | No |  |  |  |  | 0 | 0 |
|  | Ex |  |  |  |  |  | -0.6798 |
|  | Yes |  |  |  |  | -1.2344 | -0.4105 |
| Hypertension treatment | Yes |  | 0.3766 |  |  |  |  |
| Microalbuminuria |  |  |  |  |  |  |  |
| Gender | M |  |  |  |  |  |  |
|  | F |  | -0.2514 |  |  |  |  |

b) Type 2 diabetes

|  |  | No DR to Mild DR | No DR to Mod DR | No DR to RDR | Mild DR to Mod DR | Mild DR to RDR | Mod DR to RDR |
| --- | --- | --- | --- | --- | --- | --- | --- |
| Constant |  | -8.3215 | -10.9493 | -19.362 | -9.404 | -17.189 | -10.965 |
| Power |  | 0.936 | 1.161 | 1.972 | 0.889 | 1.604 | 1.140 |
| HbA1C |  | 0.1785 | 0.2657 | 0.4287 | 0.2595 | 0.4521 | 0.3094 |
| SBP |  | 0.0129 | 0.0081 | 0.0149 | 0.0128 | 0.0210 | 0.0094 |
| DBP |  | -0.0075 |  |  |  |  |  |
| BMI |  | -0.0105 | -0.0127 | -0.0131 |  |  | -0.0340 |
| Time since diagnosis | <3 |  |  | 0 |  |  | 0.0136 |
|  | 3-6 | 0.2025 | 0.2483 | 0.2639 |  |  | 0.0721 |
|  | >6 | 0.4118 | 0.4954 | 0.5686 | -0.0139 | -0.0232 | -0.0112 |
| Insulin | Yes | 0.3678 | 0.4630 | 0.1695 |  |  |  |
| Metformin | Yes | 0.0875 | 0.1213 | 0.1889 | 0.2415 | 0.0921 | 0.1796 |
| Sulfonyurea | Yes | 0.1283 | 0.1568 | 0.1224 | 0.6546 | 0.5221 | 0.5288 |
|  | No |  |  |  | 0.4263 | 0.2525 | 0.4758 |
|  | Ex |  |  |  | 0.1845 | 0.1179 | 0.2132 |
|  | Yes |  |  | -0.1059 | 0.2270 | 0.1607 | 0.2498 |
| Hypertension treatment | Yes |  | -0.0751 |  |  |  |  |
| Microalbuminuria |  | 0.0927 |  |  |  |  | -0.1685 |
| Gender | M |  |  |  |  |  | -0.1058 |
|  | F |  | -0.0623 | 0.1612 | -0.1488 | -0.1790 | -0.1697 |

Supplementary Table 3: Initial transition probability – note dynamic model

1. Type 1

|  | No DR | Mild DR | Mod DR | RDR |
| --- | --- | --- | --- | --- |
| No DR | 0.8563 | 0.1120 | 0.0305 | 0.0013 |
| Mild DR | 0 | 0.9923 | 0.0046 | 0.0031 |
| Mod DR | 0 | 0 | 0.9114 | 0.0886 |
| RDR | 0 | 0 | 0 | 1 |

1. Type 2

|  | No DR | Mild DR | Mod DR | RDR |
| --- | --- | --- | --- | --- |
| No DR | 0.9229 | 0.0611 | 0.0155 | 0.0005 |
| Mild DR | 0 | 0.9447 | 0.0533 | 0.0021 |
| Mod DR | 0 | 0 | 0.9738 | 0.0262 |
| RDR | 0 | 0 | 0 | 1 |

**Supplementary Table 4: Treatment approach**

a) First year of treatment

| **RDR state** | **Initial treatment** | **Follow up treatment (second 6 monthly cycle)** |
| --- | --- | --- |
| Pre proliferative | Screening | Screening |
| Maculopathy | Screening with OCT | Screening with OCT |
| pre proliferative and Maculopathy | Screening with OCT for 80%, Anti-VEGF for 20% @ 3* (drug + administration) | Screening with OCT for 80%, Anti-VEGF for 20% @ 2* (drug + administration) |
| Easy to treat proliferative | Three screening events + Focal Laser/PRP laser | Screening |
| easy to treat proliferative and maculopathy | Three screening events with OCT, Focal Laser/PRP laser for 60%, additional Anti-VEGF for 40% @ 3* (drug + administration) | Screening with OCT for 60%, Anti-VEGF for 40% @ 2* (drug + administration) |
| High risk Proliferative | Three screening events, Focal/PRP laser, 3 courses of laser photocoagulation | Three screening events, Focal/PRP laser, 3 courses of laser photocoagulation |
| High risk Proliferative and maculopathy with Visual impairment | Three screening events, Focal/PRP laser, 3 courses of laser photocoagulation for 60%, additional Anti-VEGF for 40% @ 3* (drug + administration) | Three screening events, Focal/PRP laser, 3 courses of laser photocoagulation for 60%, additional Anti-VEGF for 40% @ 2* (drug + administration) |
| Severe Proliferative | Vitrectomy with two screening events | Screening |
| Severe proliferative with maculopathy and vision loss | Vitrectomy with two screening events for 60%, additional Anti-VEGF for 40% @ 3* (drug + administration) | Screening with OCT for 60%, Anti-VEGF for 40% @ 2* (drug + administration) |
| SLV | Sight loss | Sight loss |

b) Second year and continuation of treatment

| **RDR state** | **Third phase treatment (Third 6 monthly cycle)** | **Continuation treatment (extending to each subsequent 6 monthly cycle)** |
| --- | --- | --- |
| Pre proliferative | Screening | Screening |
| Maculopathy | Screening with OCT | Screening with OCT |
| pre proliferative and Maculopathy | Screening with OCT for 80%, Anti-VEGF for 20% @ 2* (drug + administration) | Screening with OCT |
| Easy to treat proliferative | Screening | Screening |
| easy to treat proliferative and maculopathy | Screening with OCT for 60%, Anti-VEGF for 40% @ 2* (drug + administration) | Screening with OCT |
| High risk Proliferative | Screening | Screening |
| High risk Proliferative and maculopathy with Visual impairment | Screening with OCT for 60%, additional Anti-VEGF for 40% @ 2* (drug + administration) | Screening with OCT |
| Severe Proliferative | Screening | Screening |
| Severe proliferative with maculopathy and vision loss | Screening with OCT 60%, additional Anti-VEGF for 40% @ 2* (drug + administration) | Screening with OCT |
| SLV | Sight loss | Sight loss |

Supplementary Table 5: Initial and progressed distribution

|  | Type 1 | | Type 2 | |
| --- | --- | --- | --- | --- |
|  | Current | 2 year | Current | 2 year |
| Pre proliferative | 37.24 | 35.22 | 33.19 | 31.39 |
| Maculopathy | 20.92 | 20.04 | 24.81 | 23.76 |
| pre proliferative and Maculopathy | 15.85 | 15.45 | 18.85 | 18.25 |
| Easy to treat proliferative | 10.13 | 8.67 | 6.68 | 6.06 |
| easy to treat proliferative and maculopathy | 7.81 | 5.10 | 9.29 | 6.02 |
| High risk Proliferative | 2.94 | 5.57 | 1.94 | 4.69 |
| High risk Proliferative and maculopathy with Visual impairment | 2.26 | 4.51 | 2.69 | 4.66 |
| Severe Proliferative (vitrectomy) | 1.47 | 2.17 | 0.97 | 1.70 |
| Severe proliferative with maculopathy and vision loss | 1.13 | 2.02 | 1.35 | 2.18 |
| Blindness | 0.26 | 1.25 | 0.23 | 1.30 |
